# PCR-based syndromic tests for antibiotic stewardship in non-ventilated patients with hospital-acquired pneumonia: a multicentre randomized controlled trial

**DOI:** 10.1093/jacamr/dlag154

**Published:** 2026-07-30

**Authors:** S Kernéis, E Canouï, L Deconinck, M Dlela, S Valade, B Lortat-Jacob, C Ollivier, L Kardas-Sloma, J Charpentier, J Loubinoux, B Pilmis, A Mizrahi, R Lepeule, J W Decousser, B Berçot, C Poyart, F X Lescure, P Montravers, E Azoulay, I Durand-Zaleski, H Abdoul, J F Timsit, L Armand-Lefevre, The SHARP study group, C Alauzet, S Alviset, M Belan, S Benghanem, A Charmillon, A Contejean, V Fihman, N Gastli, C Kepeklian, A Le Monnier, A Lozniewski, N Marin, G Masson, J P Mira, P L Woerther, K Zarca

**Affiliations:** Université Paris Cité, INSERM, IAME, Paris F-75018, France; Equipe de Prévention du Risque Infectieux (EPRI), AP-HP, Hôpital Bichat, Paris F-75018, France; Equipe Mobile D’Infectiologie, AP-HP, Hôpital Cochin, Paris F-75014, France; Equipe Mobile D’Infectiologie, AP-HP, Hôpital Cochin, Paris F-75014, France; Service de Maladies Infectieuses et Tropicales, AP-HP, Hôpital Bichat, Paris F-75018, France; Médecine Intensive Réanimation, AP-HP, Hôpital Bichat, Paris F-75018, France; Médecine Intensive Réanimation, AP-HP, Hôpital Saint Louis, Paris F-75010, France; Département D’Anesthésie Réanimation AP-HP, Hôpital Bichat, Paris F-75018, France; Unité de Recherche Clinique Necker Cochin, AP-HP, Université Paris Cité, Paris, France; Unité de Recherche Clinique en économie de la Santé D'Île-de-France, DRCI, Assistance Publique-Hôpitaux de Paris, Paris 75004, France; Médecine Intensive Réanimation, AP-HP, Hôpital Cochin, Paris F-75014, France; Laboratoire de Bactériologie, AP-HP, Hôpital Cochin, Paris F-75014, France; Faculté de Médecine, Université Paris Cité, Paris, France; Clinical Microbiology Department, Hôpitaux Saint-Joseph & Marie-Lannelongue, Paris, France; Micalis Institute, UMR 1319, Université Paris-Saclay, INRAE, AgroParisTech, Orsay, France; Clinical Microbiology Department, Hôpitaux Saint-Joseph & Marie-Lannelongue, Paris, France; Micalis Institute, UMR 1319, Université Paris-Saclay, INRAE, AgroParisTech, Orsay, France; Unité Transversale de Traitement des Infections, Département de Prévention, Diagnostic et Traitement des Infections, AP-HP, Hôpital Henri Mondor, Créteil, France; Laboratoire et Equipe Opérationnelle D’Hygiène, AP-HP, Hôpital Henri Mondor, Créteil, France; Université Paris Cité, INSERM, IAME, Paris F-75018, France; Laboratoire de Bactériologie, AP-HP, Hôpital Saint Louis, Paris F-75010, France; Laboratoire de Bactériologie, AP-HP, Hôpital Cochin, Paris F-75014, France; Faculté de Médecine, Université Paris Cité, Paris, France; Université Paris Cité, INSERM, IAME, Paris F-75018, France; Service de Maladies Infectieuses et Tropicales, AP-HP, Hôpital Bichat, Paris F-75018, France; Département D’Anesthésie Réanimation AP-HP, Hôpital Bichat, Paris F-75018, France; Faculté de Médecine, Université Paris Cité, Paris, France; Médecine Intensive Réanimation, AP-HP, Hôpital Saint Louis, Paris F-75010, France; Université Paris Cité, INSERM, UMR 1342 (ICU-PEOPLE), Paris F-75010, France; Unité de Recherche Clinique en économie de la Santé D'Île-de-France, DRCI, Assistance Publique-Hôpitaux de Paris, Paris 75004, France; Unité de Recherche Clinique Necker Cochin, AP-HP, Université Paris Cité, Paris, France; Université Paris Cité, INSERM, IAME, Paris F-75018, France; Médecine Intensive Réanimation, AP-HP, Hôpital Bichat, Paris F-75018, France; Université Paris Cité, INSERM, IAME, Paris F-75018, France; Laboratoire de Bactériologie, AP-HP, Hôpital Bichat, Paris F-75018, France

## Abstract

**Objectives:**

To evaluate whether syndromic multiplex PCR (mPCR) with expert guidance improves antibiotic stewardship in patients with hospital-acquired pneumonia (HAP).

**Methods:**

We conducted a multicentre, open-label randomized controlled trial across seven French tertiary hospitals. Adults with non-ventilator-associated HAP in intensive care unit (ICU) or non-ICU wards were randomized (1:1) to conventional microbiology or mPCR testing. Empirical therapy was clinician-guided. In the intervention group, treatment was subsequently adapted according to mPCR results with expert advice. The primary endpoint was duration of broad-spectrum antibiotics (days of therapy/100 patient-days) in patients alive at end of follow-up. Secondary outcomes included appropriateness of antibiotic therapy, adverse outcomes, length of stay and costs.

**Results:**

From February 2020 to August 2023, 116 patients were randomized and 109 included in follow-up (mPCR *n* = 55; control *n* = 54). Recruitment was disrupted by the COVID-19 pandemic, leading to early trial termination at half of the planned sample size. Median age was 66 years (IQR: 55–76), 66% patients were in ICU. Mean duration of broad-spectrum antibiotics was 37.6 days of therapy/100 patient-days (standard deviation, SD 44.1) in the mPCR group versus 48.7 (SD 54.5) in controls (*P* = 0.41). mPCR significantly increased appropriate antibiotic therapy at day 0 (47% versus 24%, *P* = 0.01). No differences were evidenced in adverse outcomes, length of stay or costs.

**Conclusions:**

In this prematurely discontinued trial, mPCR with expert guidance did not reduce broad-spectrum antibiotic exposure, but improved early appropriateness of antibiotic therapy in HAP. These findings highlight the potential role of rapid diagnostics in optimizing initial antibiotic decisions, while underscoring challenges of demonstrating reductions in antibiotic consumption.

## Introduction

Respiratory tract infections account for nearly 25% of all hospital-acquired infections, and >50% of antibiotics prescribed in hospitals in the USA and Europe.^1^ Hospital-acquired pneumonia (HAP) is defined as an episode of pneumonia not associated with mechanical ventilation.^2^ The incidence of HAP is estimated to range from 1 to 10 per 1000 hospital admissions.^1,3–7^ Serious complications can occur, especially in HAP acquired in the intensive care unit (ICU HAP), where the mortality rate approaches that of ventilator-associated pneumonia (VAP).^6,8,9^ Current recommendations for HAP state that an attempt should be made to obtain respiratory samples, and strongly advise to de-escalate the antibiotic regimen on the basis of culture results.^2,10,11^ However, microbiological specimens are difficult to collect in non-intubated patients, and conventional culture has limited sensitivity and a long turnaround time. Currently, most patients are treated empirically with broad-spectrum antibiotics, and the antibiotic regimen is estimated to be inadequate in up to 70% of HAP patients.^7^

Multiplex PCR syndromic tests (mPCR) allow rapid detection of viral and bacterial pathogens, and of targeted resistance genes. In HAP and VAP, mPCR decrease the time until appropriate therapy, but interventional studies failed to show any significant impact on clinical cure, length of stay and mortality.^12–18^ Randomized trials mostly included VAP and were based on invasive sampling,^15^ which is not current practice in non-intubated patients.

This randomized controlled trial tests the hypothesis that a strategy combining rapid mPCR testing with antimicrobial stewardship reduces broad-spectrum antibiotic use without compromising patient safety in HAP patients, both in ICUs and non-ICU hospital wards.

## Methods

### Study design and participants

This randomized controlled multicentric open-label trial was conducted in eight tertiary care centres in France (of which one finally declined participation). Non-ventilated patients aged 18 years or older, with criteria of HAP and who were hospitalized for ≥48 hours at the time of pneumonia onset were included. The suspicion of pneumonia was based on the presence of new lung infiltrate on a chest X-ray or CT scan, and at least one of the following: new onset of fever (>38.5°C), purulent sputum, leucocytosis or hypoxaemia.^2,11^ Since the clinical presentation, epidemiology and therapeutic management are very specific in patients with severe chronic bronchitis structural changes, we excluded patients very severe COPD (Global initiative for chronic Obstructive Lung Disease GOLD 4) or cystic fibrosis. Patients with radiological evidence of thoracic empyema or pulmonary abscess, or those intubated at time of pneumonia onset, were also considered non-eligible. Other non-eligibility criteria included: mPCR performed before screening, inability to obtain a respiratory sample, life expectancy <90 days and non-affiliation to the national health insurance. Patients were recruited from all medical, surgical wards and ICUs of participating centres. Eligible ICU patients unable to consent immediately were included by the investigators according to a predefined emergency procedure. Pursuance of consent of either the participant or a relative (trusted person, close family) was sought as early as possible. The study protocol was approved by the Comité de Protection des Personnes Ile de France VI (Reference number: CPP 44–19).

### Randomization and masking

Participants were screened by trained study physicians. After informed consent, patients were randomly assigned (1:1) to either the control or the intervention group via the e-CRF website (electronic case report form on Cleanweb, Telemedecine), with a fixed block size of six. Randomization was stratified on the centre and on the ward (ICU versus non-ICU) at time of the inclusion. The attending physicians were not masked to patient randomization.

### Procedures

In the control group (conventional microbiology), management of HAP patients were done in accordance with international^2,10^ and national guidelines.^11^ All routine biological tests could be performed (i.e. blood cultures, direct examination and conventional culture of respiratory samples, urinary *Legionella* and *Pneumococcus* antigen tests, viral PCR tests on nasopharyngeal swabs), at discretion of the treating physician. In the intervention group (mPCR), in addition to conventional microbiology, the diagnostic strategy included a mPCR test on a respiratory sample.

To be as close as possible to real-life conditions of use, respiratory specimens were collected according to local practices. In conscious patients, samples consisted of spontaneous expectoration or sputum induction. If intubation was decided due to severity criteria, samples were collected either by endotracheal aspiration (ETA), bronchoalveolar lavage (BAL) or mini bronchoalveolar lavage (miniBAL). Respiratory specimens were analysed in each local microbiology laboratory according to current recommendations of the French Standard Guidelines in Medical Microbiology (REMIC). In brief, respiratory samples were diluted according to sample types. Then, they were streaked on recommended plates according to semi-quantitative patterns and incubated for 2 days in anaerobic, aerobic and CO_2_ conditions. Results of culture were expressed in colony-forming units (cfu)/mL. The thresholds for positivity on culture were ≥ 10^3^ cfu/mL for miniBAL, ≥ 10^4^ cfu/mL for BAL, ≥ 10^5^ cfu/mL for ETA and ≥ 10^7^ cfu/mL for sputum.^19^ Diagnostic tests for viruses and atypical bacteria were conducted if requested by the treating physician. The mPCR test was the BIOFIRE^®^ FILMARRAY^®^ Pneumonia, used according to the manufacturer’s instructions. This panel allows detection of a selected list of 26 respiratory pathogens (18 bacteria, eight viruses) and seven antibiotic resistance genes. The system integrates sample preparation, nucleic acid extraction and purification, amplification, detection and analysis, with a total run time of about 1 hour. Results of the syndromic mPCR are expressed as semi-quantitative results (10^4^ to ≥ 10^7^) in DNA-copies/mL for commonly culturable bacteria and as qualitative results (presence/absence) for resistance genes, viruses and atypical bacteria. The limit of detection for this test reported by the manufacturer is of 103.5 copies/mL.

In both groups, patients received first-line antibiotic therapy as clinically indicated by the treating physician. According to national guidelines, timing of pneumonia onset, risk factors for infection with multidrug resistant organisms, patient’s comorbidities and severity were taken into account to choose the first-line antibiotic regimen (piperacillin-tazobactam, cefepime, ceftazidime or meropenem, in monotherapy). In the intervention group, following result of the mPCR test, prescription guidance was provided by an infectious diseases specialist and/or an expert clinical microbiologist guided by a predefined decision guideline adapted from local protocols of participating centres (Table S1, available as Supplementary data at *JAC-AMR* Online). The final decision to follow this recommendation was at the treating physician’s discretion.

Follow-up consisted of three visits: at baseline (on day of randomization), on day 3 (±2 days) and on day 30 after randomization. Patients discharged before the third visit were also assessed by either telephone interview or by consultation of their medical records. On each visit, clinical parameters, antibiotic therapy, additional supportive measures (mechanical ventilation, vasopressive drugs), adverse events, radiological examinations and laboratory tests were collected.

### Outcomes

The primary outcome was the duration of broad-spectrum antibiotic therapy, expressed as the number of days of therapy (DOT) for 100 patient-days, at day 30 or end of follow-up. DOT were summed up, divided by the number of observation days and multiplied by 100. Broad-spectrum therapies were defined as follows: beta-lactams ranked ≥4 in the classification of Weiss *et al*.^20^ (piperacillin/tazobactam, cefepime, ceftazidime, carbapenems); other cephalosporins and associations (ceftaroline, ceftobiprole, ceftolozane/tazobactam, ceftazidime/avibactam, cefiderocol); therapies combining beta-lactams with either macrolides (spiramycin, azithromycin); oxazolidinones (linezolid, tedizolid); fluoroquinolones (levofloxacin, ciprofloxacin, ofloxacin, moxifloxacin); glycopeptides (vancomycin, teicoplanin) or colistin. Secondary outcomes were the duration of antibiotics, of all antimicrobial agents (both calculated in DOT for 100 patient-days as for the primary outcome), time to first modification of the antibiotic regimen, rate of de-escalation and escalation, rate of appropriate antibiotic therapy, incidence of negative outcomes (intubation for invasive ventilation, introduction of inotropes and/or vasopressor drugs, transfer to the ICU, death), of *Clostridioides difficile* colitis, in-hospital length of stay and costs. To account for deaths occurring before day 30, we also compiled the number of antibiotic-free days, calculated as the number of days alive and not receiving any antibiotic in the time period from the day of inclusion to 30 days. De-escalation was defined as downgrading from a broad spectrum to a narrower-spectrum agent within the same antibiotic class (based on Weiss *et al*. for beta-lactams^20^), changing a broad-spectrum antibiotic to a narrower-spectrum antibiotic of a different class, or discontinuing one or more drugs of a combined regimen.^21^ Antimicrobial therapy was considered appropriate if active *in vitro* against the organism(s) and proportionate (not excessively broad spectrum for the pathogen(s) identified).^16^ In both groups, the rate of appropriate antibiotic therapy was retrospectively evaluated on definitive culture results (obtained after 48 hours), and by considering micro-organisms detected above significant thresholds (results of the mPCR were not taken into account). If several respiratory samples were available for the same patient, we prioritized results of invasive samples (miniBAL or BAL over ETA over sputum). In patients with negative culture, considering that all included patients had clinical and radiological criteria of pneumonia, amoxicillin, amoxicillin/clavulanic acid, cefotaxime or ceftriaxone was considered appropriate. Number of days on broad-spectrum antibiotic therapies, nature of first modification (de-escalation, escalation) and rate of appropriate antibiotic therapy were assessed by an independent committee panel (S.K., E.C., L.D.), masked to the patient randomization group.

### Statistical analysis

According to a preliminary evaluation led in our centre over 68 episodes of HAP,^12^ duration of broad-spectrum antibiotics was estimated at ∼2.9 DOT. Previous studies^22^ estimated that the mPCR could lead to a 2-day reduction in duration of broad-spectrum antibiotics, which was considered as clinically relevant. With a power of 80% and an estimated SD of 5, a sample size of 200 participants was necessary.

However, the study started in February 2020 and inclusions were strongly disrupted by COVID-19 response as all study investigators were infectious diseases or intensive care physicians. Despite several prolongations of the inclusion period, and due to resource constraints, the study had to be terminated in August 2023, reaching about half of the inclusion targets.

As predefined in the protocol, the primary outcome (number of days on broad-spectrum antibiotics) was compared between control and mPCR groups, excluding patients deceased before day 30 [modified intention-to-treat (mITT) analysis]. The study was initially designed to include patients outside the ICU, with life expectancy >90 days, we then expected few deaths before day 30 and considered that mITT analysis was clinically acceptable. A sensitivity analysis of the primary outcome was secondly performed on the full analysis set (ITT population). Secondary outcomes were analysed in the ITT population. A subgroup analysis was performed on patients who acquired HAP in the ICU. Quantitative outcomes were compared between groups using the Wilcoxon rank-sum test, as the normality assumption was rejected by the Shapiro–Wilk test (*P* < 0.0001). Unadjusted treatment effects were estimated using the Hodges–Lehmann shift estimator with 95% CI. All analyses were subsequently adjusted for centre and ICU status using a stratified Wilcoxon test (van Elteren test) and a rank-based ANCOVA to obtain adjusted treatment effect estimates with 95% CIs. For categorical secondary outcomes, analyses were not stratified due to the small number of events, particularly in centres where the event count was near zero. Stratification or multivariate adjustment would have led to unreliable estimates. These outcomes were compared using Pearson’s *χ*^2^ test, or Fisher’s exact test when expected cell counts were fewer than five. The effect size was estimated using the risk difference along with its 95% CI. No imputation of missing data was performed. The primary outcome was available for all patients included in the modified ITT analysis, as was the case for the main secondary outcomes assessed in the full ITT population. For the other secondary outcomes, a small number of data points were missing. A complete case analysis was therefore performed throughout. All tests were bilateral with a significance level of 5%. Quantitative data are expressed as either median with IQR or mean with standard deviation (SD), and qualitative data as *n* (percentage). SAS (version 9.4) was used for the statistical analysis.

### Costs analysis

We performed a cost-consequences analysis. The cost perspective was limited to the hospital. Hospital costs were estimated for each patient over the 30-day follow-up period. We compared costs of production of hospital stay, antimicrobial treatment and costs linked to the mPCR test itself on both groups. For hospital stays, we used ICD-10 codes for pneumonia and the DRG (Diagnosis Related Group) related to these codes. We weighted the DRG daily costs by their relative frequency and adjusted them to the length of stay. To reflect actual hospital costs of patient’s antimicrobial treatment, we extracted the price of each International Non-proprietary named drug molecule from the database of drug costs at our institution multiplied by its consumption as recorded in the eCRF. Costs of the mPCR test included equipment and maintenance fees and the laboratory technician time-related salary. Differences between groups were estimated using non-parametric bootstrap approach with 2000 replications. The bootstrap method was chosen because patient cost data were highly skewed and contained extreme values, which can violate the assumptions of traditional parametric tests. One-way sensitivity analyses were conducted to assess the robustness of the cost-consequences results. Two parameters were varied independently: (i) the distribution of DRGs used to estimate the theoretical daily hospitalization cost and (ii) the unit price of the mPCR test. For DRGs, minimum and maximum plausible values were applied, while for the mPCR test, the unit price was varied by ±20%. Results were displayed in a tornado diagram showing the deviation in total cost difference from the base-case estimate. R version 4.3.2 was used for the statistical analysis.

This trial was registered with the International Clinical Trials Registry Platform under reference NCT04153682.

## Results

**Figure 1.**
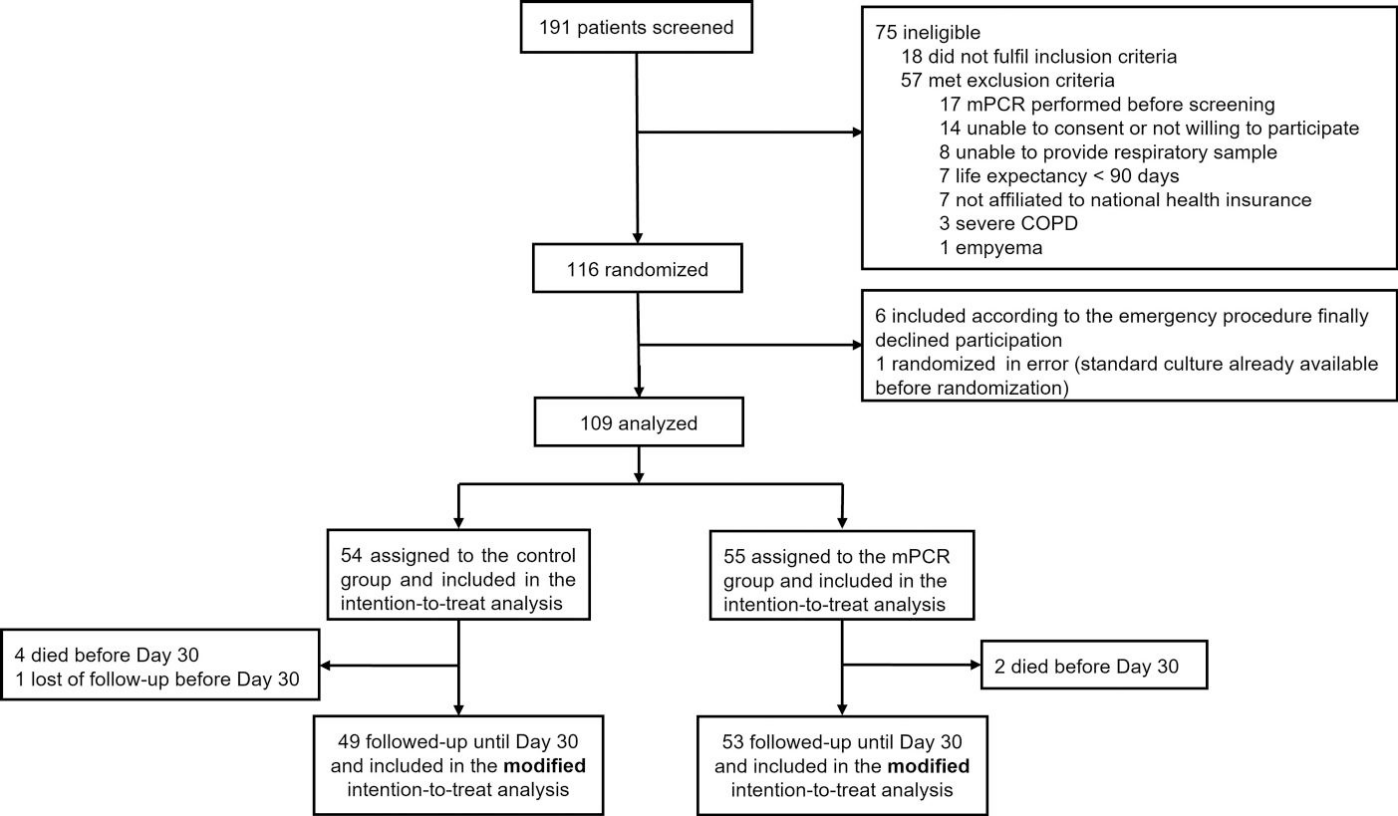
Flowchart of study participants.

Between 21 February 2020 and 21 August 2023, 191 patients with HAP were screened for eligibility and 116 were randomized (Figure 1). Seven participants were subsequently excluded (six were included according to the emergency procedure but finally declined participation, and one was randomized in error). Overall, 55 participants assigned to the mPCR group and 54 to the conventional microbiology group were included in the ITT population (Figure 1).

Baseline demographics were similar between the two groups (Table 1). Median age was 66.0 years (IQR: 55.0, 76.0) and 68 (62%) were male. Twenty-six (28%) had immune suppression and 20 (18%) chronic obstructive pulmonary disease. MDRO carriage was detected in the previous 12 months in 15 (14%) participants: ESBL-producing Enterobacterales (ESBL-E) in nine (8%) and MRSA in six (6%). Most (64, 60%) had received antibiotics in the past 6 months. At randomization, 72 (66%) patients were in ICU, 23 (21%) needed either non-invasive ventilation or nasal high flow (HNF) therapy. According to inclusion criteria, no patient was on mechanical ventilation at inclusion, but 30 (28%) were intubated within 24 hours after randomization. On thoracic imaging, 90 (83%) had bilateral images of pneumonia (consolidation or interstitial pattern), and 39 (36%) pleural effusion.

**Table 1.** Baseline characteristics of patients

	mPCR group (*N* = 55)	Missing	Control group (*N* = 54)	Missing	Total (*N* = 109)
Age, years	67 (54, 77)	0	66 (55, 74)	0	66 (55, 76)
Males	37 (67%)	0	31 (57%)	0	68 (62%)
Coexisting conditions					
Charlson Comorbidity Index	5 (4, 7)	0	5 (3, 7)	0	5 (3, 7)
Immunosuppression^a^	14 (31%)	10	12 (26%)	7	26 (28%)
Chronic obstructive pulmonary disease	9 (16%)	0	11 (20%)	0	20 (18%)
Current or past smoker	37 (72%)	4	28 (57%)	5	65 (65%)
Risk factors of MDRO^b^ carriage at inclusion					
Current residence outside metropolitan France	2 (4%)	0	0	0	2 (2%)
Antibiotics in the past 6 months	35 (64%)	0	29 (56%)	2	64 (60%)
Known carriage of MDRO in the past 12 months		0		0	
ESBL-E	5 (9%)		4 (7%)		9 (8%)
MRSA	3 (6%)		3 (6%)		6 (6%)
CPE	2 (4%)		2 (4%)		4 (4%)
CRAB	1 (2%)		0		1 (1%)
Clinical status		0		0	
ICU	34 (62%)		38 (70%)		72 (66%)
Highest heart rate, beats per min	101 (90, 120)	4	102 (90, 114)	5	102 (90, 120)
Lowest systolic blood pressure, mmHg	107 (93, 124)	3	104 (88, 116)	3	106 (90, 120)
Neurological impairment	13 (24%)	0	5 (9%)	1	18 (17%)
Septic shock	9 (16%)	0	9 (17%)	0	18 (17%)
Vasopressive drugs on day of randomization	16 (29%)	0	11 (20%)	0	27 (25%)
Respiratory assistance					
Non-invasive ventilation at inclusion	3 (5%)	0	7 (13%)	0	10 (9%)
Nasal high flow therapy at inclusion	4 (7%)	0	9 (17%)	0	13 (12%)
Intubation on day of randomization^c^	17 (31%)	0	13 (24%)	0	30 (28%)
Haemodialysis	4 (7%)	1	5 (9%)		9 (8%)
Biological parameters					
Creatinin, mmol/L	100 (59, 154)	0	84 (51, 176)	0	96 (56, 157)
White blood cell count, ×10^9^/L	10.8 (6.9, 13.7)	0	10.3 (7.1, 13.3)	0	10.6 (7, 13.5)
C-reactive protein, mg/L	159 (115, 195)	24	108 (68, 174)	20	141 (79, 190)
Procalcitonin, μg/L	0.5 (0.2, 1.6)	39	0.5 (0.3, 3)	29	0.5 (0.2, 2.7)
Imaging (chest X-ray or CT scan)					
Bilateral images	42 (78%)	1	48 (89%)	0	90 (83%)
Pleural effusion (minimal or moderate)	20 (37%)	1	19 (35%)	0	39 (36%)

Data are *n* (%) or median (IQR).

^a^Immunocompromising conditions included: haematological disease, HIV, solid organ transplantation, stem cell transplantation in the past 5 years and treatment with any immune suppressive agent in the past 3 months; ^b^MDRO: Multidrug Resistant Organisms (CRAB: Carbapenem-producing *Acinetobacter baumannii*, ESBL-E: ESBL-producing Enterobacterales, CPE: Carbapenemase-producing Enterobacterales, ^c^According to inclusion criteria, no patients were intubated at time of randomization, but 30 required intubation and mechanical ventilation on the same day.

One hundred and ninety-two respiratory samples were performed: sputum (47, 24%), ETA (eight, 4%), BAL (123, 64%) or miniBAL (14, 7%). Micro-organisms detected in both groups are presented in Table 2. The overall rate of documentation was of 64% (35/55) in the intervention group, compared with 31% (17/54) in the control group. The most commonly bacteria identified were *Pseudomonas aeruginosa* (16/109, 15%), *Escherichia coli* (10/109, 9%) and *Haemophilus influenzae* (10/109, 9%). ESBL-E were detected in four (4%) patients. No atypical bacteria (*Chlamydia pneumoniae*, *Mycoplasma pneumoniae* and *Legionella pneumophila*) were identified. Overall, viral respiratory tests were positive in 24 patients, mainly SARS-CoV-2 (*n* = 10), coronavirus (*n* = 7), human rhino/enterovirus (*n* = 6) and influenza A (*n* = 5).

**Table 2.** Micro-organisms detected in both groups

	Total (*N* = 109)	Control group (*N* = 54)	mPCR group (*N* = 55)			
			mPCR (*N* = 55)	Routine microbiology (*N* = 55)	Overall (*N* = 55)	Concordance
Bacteria						
* Pseudomonas aeruginosa*	16 (15%)	6 (11%)	9	7	10 (18%)	51 (92.7%)
* Escherichia coli*	10 (9%)	1 (2%)	8	5	9 (16%)	50 (90.9%)
* Haemophilus influenzae*	10 (9%)	2 (4%)	8	3	8 (15%)	50 (90.9%)
* Enterobacter cloacae*	8 (7%)	2 (4%)	5	2	6 (11%)	50 (90.9%)
* Staphylococcus aureus*	7 (6%)	3 (5%)	3	3	4 (7%)	53 (96.4%)
* Klebsiella pneumoniae*	6 (6%)	3 (5%)	2	3	3 (5%)	54 (98.2%)
* Moraxella catarrhalis*	4 (4%)	0	4	2	4 (7%)	53 (96.4%)
* Serratia marcescens*	4 (4%)	2 (4%)	2	1	2 (4%)	54 (98.2%)
* Klebsiella oxytoca*	3 (3%)	0	3	0	3 (5%)	52 (94.5%)
* Streptococcus pneumoniae*	3 (3%)	0	3	2	3 (5%)	53 (96.4%)
* Acinetobacter baumannii complex*	2 (2%)	1 (2%)	1	0	1 (2%)	54 (98.2%)
* Klebsiella aerogenes*	1 (1%)	1 (2%)	0	0	0	55 (100%)
* Proteus* spp.	1 (1%)	0	1	0	1 (2%)	54 (98.2%)
* Streptococcus agalactiae*	1 (1%)	0	1	0	1 (2%)	54 (98.2%)
Hafnia alvei*	1 (1%)	0	NA	1	1 (2%)	NA
Burkholderia cenocepacia*	1 (1%)	1 (2%)	NA	0	0	NA
Mycobacterium xenopi*	1 (1%)	0	NA	1	1 (2%)	NA
Stenotrophomonas maltophilia*	1 (1%)	0	NA	1	1 (2%)	NA
Nocardia*	1 (1%)	0	NA	1	1 (2%)	NA
Antimicrobial resistance						
MRSA	0	0	0	0	0	100%
ESBL-E	4 (4%)	1 (2%)	3	1	3 (5%)	96.4%
Viruses						
SARS-CoV-2*	10 (10%)	7 (13%)	NA	3	3 (5%)	NA
Coronavirus	7 (6%)	3 (6%)	0	4	4 (7%)	88.6%
Human Rhinovirus/Enterovirus	6 (6%)	2 (4%)	4	2/2^a^	4 (7%)	100%
Influenza A	5 (5%)	2 (4%)	3	1/1^b^	3 (5%)	100%
HSV*	2 (2%)	1 (2%)	NA	1	1 (2%)	NA
Respiratory Syncytial virus	1 (1%)	0	1	1	1 (2%)	100%
Bocavirus*	1 (1%)	0	NA	1	1 (2%)	NA

Overall, 192 respiratory samples were performed during the study period.

For mPCR, no consensual thresholds are defined, then all positive results are reported. For culture, thresholds for positivity were ≥ 10^3^ cfu/mL for miniBAL, ≥ 10^4^ for BAL, ≥ 10^5^ for ETA and ≥ 10^7^ for sputum. In the mPCR group, micro-organisms are those detected either by mPCR or routine biological tests (culture, urinary antigen tests, viral PCR tests). In the control group, micro-organisms are those detected by routine biological tests only. If several respiratory samples were available for the same patient, we prioritized the results of invasive samples (miniBAL or BAL over ETA over sputum).

Micro-organisms marked with * are not detected by the panel.

Concordance was assessed in the mPCR group (*n* = 55), and only for micro-organisms (bacteria and viruses) and resistance markers included in the panel. For each sample, we evaluated whether results were concordant between the mPCR and the routine biological tests (presence or absence of the pathogen and antimicrobial susceptibility). For example, *P. aeruginosa* was detected on nine samples by mPCR and on seven samples by culture. It was detected only by mPCR on three samples and only on culture on one, leading to four discordances over 55 samples (concordance: 51/55 = 92.7%).

^a^Only two mPCR-positive samples were also tested by conventional PCR test, ^b^Only 1 mPCR-positive sample was also tested by conventional PCR test. NA = not applicable.

In the intervention group (*n* = 55), respiratory samples were sputum (26, 47%), ETA (5, 9%), BAL (19, 35%) and miniBAL (5, 9%). mPCR results were communicated to the clinician 3.9 hours after sampling (median, IQR: 2.6–9.6). The mPCR detected at least one bacteria on 29/55 (53%) samples: 14/26 (54%) on sputum, 4/5 (80%) on ETA, 7/19 (37%) on BAL and 4/5 on miniBAL. Concordance between mPCR and routine microbiology tests was ≥95% for most bacteria species (Table 2). Among 35 discordances reported (in 22 patients), 29 were minor (bacteria were detected on both mPCR and culture but below significant thresholds on culture; or culture did not detect pathogens that were detected at low quantification (<10^4^) on mPCR). Details on major discordances are given in the Appendix (Table S2).

**Figure 2.**
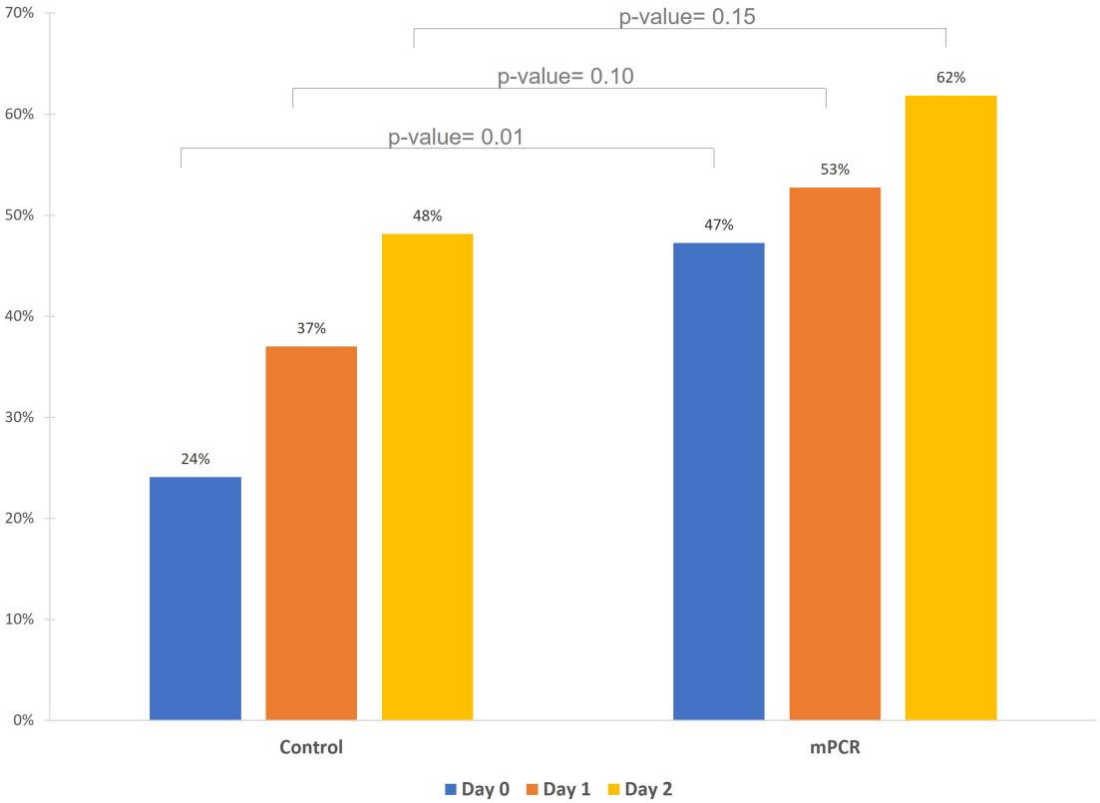
Rates of appropriate antibiotic therapy on day 0 (day of randomization), day 1 and day 2 after randomization. Antibiotic therapy was considered appropriate if active *in vitro* against the organism(s) and proportionate (not excessively broad spectrum for the pathogen(s) identified). In both groups, rates of appropriate antibiotic therapy were retrospectively evaluated on definitive culture results (obtained after 48 hours), and by considering micro-organisms detected above significant thresholds. If several respiratory samples were available for the same patient, we prioritized results of invasive samples (miniBAL or BAL over ETA over sputum). In patients with negative culture (*n* = 30), considering that all included patients had clinical and radiological criteria of pneumonia, amoxicillin, amoxicillin/clavulanic acid, cefotaxime or ceftriaxone was considered appropriate.

On day of inclusion, 104/109 participants (95%) were on antibiotics. In the modified ITT population (*n* = 102), mean duration of broad-spectrum antibiotic therapies was 37.6 DOT for 100 patient-days (SD: 44.1) in the mPCR group, and 48.7 (SD: 54.5) in the control group (*P* value = 0.41; primary outcome, Table 3). The sensitivity analysis performed on the ITT population (*n* = 109) showed similar results (*P* = 0.44, Table S3). DOT of all antibiotics, of all antimicrobial agents and of antibiotic-free days did not significantly differ between groups (ITT population). As shown in Table 3 and Figure 2, 26/55 (47%) patients in the mPCR group were receiving appropriate antibiotics by 24 hours after randomization (day 0), versus 13/54 (24%) in the control group (chi-square *P* = 0.01). Although not significantly, the rate of appropriate antibiotic therapy was also higher in the mPCR group on days 1 and 2 post-randomization. On day 2 post-randomization, 45% of patients (49/109) were receiving inappropriate therapy (either non-active and/or excessively broad) as quoted by the committee panel (Table S4).

**Table 3.** Antibiotic use

	mPCR group	Control group	Effect size [95% CI]	*P* value
Primary outcome (modified ITT analysis)	***n*** **=** **53**	***n*** **=** **49**		
Median duration of broad-spectrum antibiotics, DOT^a^	22.7 (3.7, 53.3)	26.9 (9.7, 66.7)	3.3 [−8.4 ; 15.0]	0.32^b^
Secondary outcomes (ITT analysis)	***n*** **=** **55**	***n*** **=** **54**		
Median duration of antibiotics, DOT	50.0 (23.3, 103.4)	55.8 (23.3, 133.3)	3.4 [−8.9 ; 15.8]	0.37^b^
Median duration of all antimicrobial agents^c^, DOT	59.1 (23.3, 126.7)	67.8 (23.5, 160.0)	6.5 [−9.0 ; 15.7]	0.39^b^
Median number of antibiotic-free days	53.3 (14.3, 80.0)	50.7 (0.0, 80.8)	−3.4 [−15.7 ; 8.9]	0.61^b^
Appropriate antimicrobial therapy				
on day of randomization (day 0)	26 (47%)	13 (24%)	0.23 [0.06 ; 0.41]	0.01^d^
on day 1 after randomization	29 (53%)	20 (37%)	0.16 [−0.03 ; 0.34]	0.10^d^
on day 2 after randomization	34 (62%)	26 (48%)	0.14 [−0.05 ; 0.32]	0.15^d^
De-escalation on day of randomization	14 (25%)	15 (28%)	−0.02 [−0.19 ; 0.14]	0.78^d^
Escalation on day of randomization	3 (5%)	5 (9%)	−0.04 [−0.14 ; 0.06]	0.49^e^

Data are *n* (%) or median (interquartile range).

Durations are expressed as the number of DOT for 100 patient-days, at day 30 or end of follow-up.

^a^DOT per 100 patient-days from day 0 (inclusion) to day 30 or end of follow-up, see text for details.

^b^Wilcoxon rank-sum *P* value.

^c^Including antibiotics, antivirals and antifungals.

^d^ *χ* ^2^  *P* value.

^e^Fisher exact *P* value.

There was no evidence of difference between groups on rates of escalations/de-escalations, neither on day of randomization (Table 3), nor at the end of follow-up (data not shown). The median time to first modification of the antimicrobial regimen was 1.0 (0.0, 2.0) in both groups (*P* = 0.38, Wilcoxon rank-sum test). Overall, 85 de-escalations of the antibiotic regimen were reported, 62 consisted of discontinuation of at least one antibiotic and 28 in spectrum downgrading (Table S5).

The most commonly administered antibiotics were carbapenems (meropenem and imipenem), piperacillin-tazobactam and cefotaxime (Table 4 and Figure S1). The mean number of carbapenems-free days for 100 patient-days (coupling meropenem and imipenem) was of 94.4 (SD: 24.4) in the mPCR group, and 88.6 (SD: 34.0) in the control group (Wilcoxon rank-sum *P* = 0.35).

**Table 4.** Antibiotics administered

	mPCR group (*N* = 55)	Control group (*N* = 54)	Total (*N* = 109)
Meropenem	284.5	793.8	1078.3
Imipenem	243.9	29.8	273.7
Cefotaxime	452.0	351.8	803.8
Ceftriaxone	80.1	36.1	116.2
Piperacillin-tazobactam	271.1	552.5	823.6
Amoxicillin-clavulanate	307.9	319.3	627.2
Ceftazidime	179.3	242.4	421.7
Cefepime	287.4	500.8	788.2
Cefiderocol	106.7	0	106.7
Metronidazole	218.0	313.6	531.6
Linezolid	208.2	244.6	452.8
Trimethoprime-sulfamethoxazole	140.1	210.2	350.3
Levofloxacin	203.0	45.8	248.8
Doxycyclin	111.1	73.3	184.4
Colistine-polymyxine E	100.0	0	100.0

Cumulated number of DOT for 100 patient-days, at day 30 or end of follow-up. Data are presented for the 15 most commonly prescribed antibiotics.

Seven patients in the mPCR group (13%) and six (11%) controls experienced clinical deterioration during follow-up (*P* = 0.78, Table 5). The median in-hospital stay was 25.0 days (10.0, 30.0) in the mPCR group and 18.5 days (9.0, 29.0) in the control group (*P* = 0.27, Wilcoxon rank-sum test). In ICU patients (*n* = 72), no patient of the mPCR group and 3 (20%) of the control group died during follow-up (*P* = 0.09, Table S6). On day 0, 20/34 (59%) participants in the mPCR group were receiving appropriate antibiotics, compared with 9/38 (24%) in the control group (*P* = 0.002). In non-ICU wards (*n* = 37), three (14%) participants in the mPCR group and none of the control group subsequently needed transfer to ICU (*P* = 0.13). We did not show any differences between groups neither on mortality, rates of appropriate antibiotic therapy and of escalations/de-escalations in non-ICU wards (Table S7).

**Table 5.** Outcomes (ITT analysis)

	mPCR group (*N* = 55)	Missing	Control group (*N* = 54)	Missing	Effect size [95% CI]	*P*
Clinical deterioration (composite criteria)^a^	7 (13%)		6 (11%)		0.02 [−0.10 ; 0.13]	0.78^b^
Transfer to the ICU (*n* = 37)	3	0	0	1	—	
Introduction of vasopressive drugs (*n* = 82)	1	0	4	2	—	
Intubation (*n* = 79)	1	0	2	3	—	
Death	2	0	4	0	—	
*Clostridioides difficile* colitis	0	1	2 (4%)	3	−0.04 [−0.09 ; 0.01]	0.23^c^

Data are *n* (%). Criteria for deterioration were assessed from the day following randomization (day 1) to day 30.

On day of randomization (day 0), 37 patients were outside the ICU, 82 were not receiving vasopressive drugs and 79 were not intubated.

^a^Composite criteria including transfer to the ICU, intubation, introduction of vasopressive drugs, death.

^b^ *χ* ^2^  *P* value.

^c^Fisher exact *P* value.

Overall, the average total cost per patient was €23 635 in the mPCR group and €22 802 in the control group (difference €833, 95% CI of the difference: €−5227; €7435, Table S8). The mean cost of antimicrobial treatment was €2849 in the mPCR group and €423 in the control group (95% CI of the difference €100; €6236), but higher costs in the mPCR group were driven by use of expensive antifungal drugs in five patients (of mean cost €6706). To assess the robustness of this finding, we repeated the analysis excluding these five patients; the mean antimicrobial treatment cost difference between groups was −€6 (95% CI: −€163; €160), confirming that the cost difference observed in the primary analysis was not attributable to the mPCR-guided strategy itself. One-way sensitivity analyses showed that the DRG distribution assumption had the greatest influence on the estimated difference in total cost between groups (range: €625 to €1692), while variations in the unit price of the mPCR test had minimal impact on the results range: €797 to €851, Figure S2).

## Discussion

SHARP was a pragmatic, multicentre randomized controlled trial assessing the impact of a mPCR panel in ICU and non-ICU patients with suspected HAP. The COVID-19 pandemic markedly disrupted recruitment and, despite recovery from 2022 onward, only about half of the target sample size was achieved. Despite a trend towards lower broad-spectrum antibiotic use in the intervention arm, statistical significance was not reached for the primary endpoint. mPCR, however, significantly improved the rate of appropriate antibiotic therapy 24 hours after diagnosis. No differences were evidenced in mortality, escalation or de-escalation rates, adverse clinical outcomes, length of stay or incidence of *Clostridioides difficile* infection.

SHARP was initially designed to focus on non-ICU HAP, without systematic invasive sampling, adopting a pragmatic approach reflecting a common situation responsible for substantial antibiotic use, and not previously examined in RCTs. Our hypothesis was that, beyond individual clinical benefits for patients, prompting targeted therapy could reduce the use of broad-spectrum antibiotics at the hospital level and then contribute to global antimicrobial stewardship. DOT was selected to evaluate the primary outcome, as it captures both overall exposure and appropriateness, and is not influenced by dosing adjustments. However, due to recruitment challenges, inclusion criteria were subsequently broadened to include ICU patients and deaths during follow-up may have biased DOT estimates. DOT of broad-spectrum antibiotics, of all antibiotics and of all antimicrobial agents were lower in the intervention group, but differences were small with substantial variability and the study was underpowered to draw firm conclusions.

To be considered as appropriate, the antimicrobial therapy had to be both active *in vitro* and proportionate (not excessively broad spectrum), explaining why only 47% and 24% patients were receiving appropriate antibiotics on day 0 in the mPCR and the control groups respectively. The rate of appropriate antibiotic therapy at 24 hours was significantly higher in the mPCR group, despite similar escalation and de-escalation rates. Rates of de-escalation on day of randomization (25%) were lower than expected in the intervention group.^12^ Previous randomized trials, mostly conducted in ICU populations^14–16,18^ have reported comparable findings. This may reflect a strategy in which clinicians delay antibiotic initiation while awaiting rapid results (3.9 hours in median in SHARP, 2 hours in INHALE which used point-of-care testing^16^), thereby enabling tailored therapy from the outset. At an individual level, improving early appropriateness of antibiotics is probably a clinically and ecologically relevant objective, but our study did not evidence effects on clinical outcomes or overall antibiotic exposure over the study population. Importantly, concerns that highly sensitive syndromic panels might increase inappropriate broad-spectrum antibiotic use were not supported by our findings.

Total hospital costs did not differ between groups. Costs were primarily driven by length of stay, which was similar, and by empirical use of antifungal agents in a small number of patients, all in the intervention group. As the mPCR panel did not include fungal targets, opportunities for antifungal de-escalation were limited. Given the small sample size, this imbalance probably reflects random variation rather than an effect of the intervention.

Strengths of SHARP include its randomized design, inclusion of non-ICU HAP patients, a population less frequently studied, and its pragmatic approach without systematic invasive sampling. In addition, focusing on antibiotic consumption rather than rates of escalations/de-escalations aligns with antimicrobial stewardship priorities. The study population also appears to be representative of a typical HAP population, which supports the generalizability of the findings.

The main limitation is insufficient statistical power, precluding definitive conclusions on broad-spectrum antibiotics use. Recruitment was severely affected by the COVID-19 pandemic, initially because investigators were heavily involved in clinical care and subsequently because enrolment in COVID-19-specific therapeutic research protocols was prioritized. Although enrolment resumed in 2022 in line with the anticipated recruitment trajectory, the trial was prematurely discontinued because of financial constraints. Second, the low proportion of patients excluded due to infeasible sampling suggests potential selection bias towards patients able to provide respiratory samples, limiting generalizability, particularly in non-ICU settings. Third, conversely to other RCTs, mPCR results were available only in the intervention group, and appropriateness was assessed on the basis of on culture results, which may underestimate true pathogen detection, especially after previous antibiotic exposure. Finally, the SHARP trial was conducted exclusively in France, and the level of antimicrobial resistance reported in our study is consistent with that observed in Northern European settings. However, the impact of mPCR-guided strategies may differ in settings with higher antimicrobial resistance rates.

### Conclusion

Although underpowered, mPCR combined with expert prescribing guidance improved the rate of early appropriate antibiotic therapy in HAP patients, which is an encouraging result at a patient-level. However, the effect on overall use of broad-spectrum antibiotics, and consequently the impact for global antimicrobial stewardship remains uncertain. Further research is needed to assess benefits in settings with high prevalence of antimicrobial resistance.

## Supplementary Material

dlag154_Supplementary_Data

## Transparency declarations

Laurène Deconinck declares support for attending meetings and/or travel by MSD, Gilead, Tillotts Pharma, ViiV, Eumedica, Abbvie and Shionogi. Philippe Montravers declares personal fees (consulting, or payment or honoraria for lectures, presentations, speakers bureaus, manuscript writing or educational events) and participation on a Data Safety Monitoring Board or Advisory Board from Menarini, Pfizer and Viatris. Elie Azoulay declares grants or contracts provided to his institution from MSD AVENIR and payment or honoraria for lectures, presentations, speakers bureaus, manuscript writing or educational events from Pfizer, Alexion, Gilead, Mundipharma and Fisher&Payckle. Isabelle Durand-Zaleski declares payment to her institution from the French Ministry of Health. Jean-François Timsit declares personal fees (consulting, or payment or honoraria for lectures, presentations, speakers bureaus, manuscript writing or educational events) and participation on a Data Safety Monitoring Board or Advisory Board from bioMerieux, Gilead, Pfizer, Advanz, Menarini, Merck, Pfizer, Shionogi, advanz Mundipharma, Gilead, bioMerieux and Qiagen. Laurence Armand-Lefevre declares payment to her institution from bioMerieux and support for attending meetings and/or travel from bioMerieux. The other authors declare no relationships, activities or interests.

## Supplementary data

Figures S1 and S2 and Tables S1 to S8 are available as Supplementary data at *JAC-AMR* Online.
