# Supplementary material for: PCR-based syndromic tests for antibiotic stewardship in non-ventilated patients with hospital-acquired pneumonia: a multicentre randomized controlled trial
Source: JAC Antimicrob Resist. 2026 Jul 30;8(4):dlag154. doi: 10.1093/jacamr/dlag154 (PMC13420529; doi:10.1093/jacamr/dlag154)

Online Supplement to:

**PCR-based syndromic tests for antibiotic stewardship in patients with hospital-acquired pneumonia (SHARP): a multicenter, randomized, controlled trial**

Kernéis S, Canouï E, Deconinck L, Dlela M, Valade S, Lortat-Jacob B, Ollivier C, Kardas-Sloma L, Charpentier J, Loubinoux J, Pilmis B, Mizrahi A, Lepeule R, Decousser JW, Berçot B, Poyart C, Lescure FX, Montravers P, Azoulay E, Durand-Zaleski I, Abdoul H, Timsit JF, Armand-Lefevre L and the SHARP study group

**Table of contents**

[Table S1. Algorithm 2](#_Toc234237899)

[Table S2. Discordances. 3](#_Toc234237900)

[Table S3. Sensitivity analysis of the primary outcome. 3](#_Toc234237901)

[Table S4. Inadequate therapies. 4](#_Toc234237902)

[Table S5. Escalations and De-escalations. 6](#_Toc234237903)

[Figure S6. Antibiotics at inclusion (empirical) and at day 2. 10](#_Toc234237904)

[Table S7. Subgroup analysis - ICU 11](#_Toc234237905)

[Table S8. Subgroup analysis – non-ICU wards 11](#_Toc234237906)

[Table S9. Cost-consequence analysis. 11](#_Toc234237907)

[Figure S10. Tornado diagram 12](#_Toc234237908)

# **Table** **S1. Algorithm**

Decision algorithm for adaptation of the empirical antibiotic regimen according to the mPCR test results. Patients received empirical antibiotic therapy as clinically indicated by the treating physician. According to national guidelines, timing of pneumonia onset, risk factors for infection with multidrug resistant organisms, patient’s comorbidities and severity were taken into account to choose the empirical antibiotic regimen: either amoxicillin-clavulanate or piperacillin-tazobactam in monotherapy, or in specific situations (i.e. known carriage of MDRO): cefepime, ceftazidime or meropenem in monotherapy. In the intervention group, following result of the mPCR test, prescription guidance was provided by an infectious diseases specialist and/or an expert clinical microbiologist, guided by the following decision guideline.

| **Microorganism detected by mPCR** | **Recommended antibiotic regimen** | **Alternative** |
| --- | --- | --- |
| *Escherichia coli* | Cefotaxime or Ceftriaxone | Aztreonam |
| *Proteus spp.* |  |  |
| *Klebsiella pneumoniae* |  |  |
| *Klebsiella oxytoca* |  |  |
| *Serratia marcescens* | Cefepime |  |
| *Klebsiella aerogenes* |  |  |
| *Enterobacter cloacae* |  |  |
| All *Enterobacteriacae* |  |  |
| CTX-M | Meropenem or Imipenem | Ceftazidime-avibactam |
| KPC | ATB regimen to be chosen among: Colistin / Ceftazidime-Avibactam / Aztreonam / Meropenem / Aminoglycosides / Fosfomycin | |
| Oxa48-like |  |  |
| NDM |  |  |
| *Pseudomonas aeruginosa* | Ceftazidime or Piperacillin-Tazobactam or Cefepime | Meropenem + Amikacin or Aztreonam + Amikacin |
| IMP | Aztreonam +/- Amikacin | Undefined |
| VIM |  |  |
| *Acinetobacter calcoaceticus baumannii complex* | Ceftazidime or Imipenem + Amikacin or Tobramycine | Undefined |
| *Haemophilus influenzae* | Cefotaxime or Ceftriaxone or  Amoxicillin- clavulanate | Levofloxacin if not exposed to quinolones < 3 months |
| *Moraxella catarrhalis* |  |  |
| *Staphylococcus aureus* | Cefazoline or Cloxacillin or Amoxicillin- clavulanate | Vancomycin or Linezolid |
| mecA/C and MREJ | Vancomycin or Linezolid | Undefined |
| *Streptococcus agalactiae* | Amoxicillin | Pristinamycin or Levofloxacin |
| *Streptococcus pneumoniae* | Amoxicillin | Pristinamycine or Lévofloxacine |
| *Streptococcus pyogenes* | Amoxicillin | Pristinamycine or Spiramycin or Levofloxacin |
| *Chlamydia pneumoniae* | Spiramycin or Azithromycin | Levofloxacin |
| *Legionella pneumophila* | Spiramycin or Azithromycin | Levofloxacin |
| *Mycoplasma pneumoniae* | Spiramycin | Levofloxacin |

# **Table S2. Discordances.**

Six major discordances (in 5 patients) reported between mPCR and culture results.

| **Sample** | **mPCR result** | **Culture result** |
| --- | --- | --- |
| Sputum | Test failure | >10^7^ *Staphylococcus aureus* |
| Sputum | Negative | >10^7^ *Klebsiella pneumoniae* |
| Endotracheal aspiration | *Enterobacter cloacae* >10^7^ copies/mL | Negative |
| Sputum | *Staphylococcus aureus* >10^7^ copies/mL  *Streptococcus pneumoniae* >10^7^ copies/mL | Negative |
| Sputum | *Haemophilus influenzae* >10^7^ copies/mL | Negative |

# **Table** **S3. Sensitivity analysis of the primary outcome.**

Intention-to-treat population (n=109). Durations are expressed as the number of days on therapy (DOT) for 100 patients-days, at day 30 or at the end-of follow-up.

|  | mPCR group (N=55) | Control group (N=54) | Total (N=109) | p-value |
| --- | --- | --- | --- | --- |
| Mean duration of broad-spectrum antibiotics, DOT | 23.3 (3.7, 54.8) | 28.5 (8.7, 75.8) | 25.0 (7.7, 61.5) | 0.44^1^ |

^1^Wilcoxon rank sum p-value

# **Table S4. Inadequate therapies**.

Antibiotic regimen and microbiology results in 50 patients with inadequate antibiotic regimen at Day 2, as quoted by the committee panel. NPS : Nasopharyngeal swab ; WT : wild type ; HP : High production ; ETA : endotracheal aspiration, BAL : bronchoalveolar lavage ; miniBAL : mini bronchoalveolar lavage ; MSSA : Methicillin-susceptible *Staphylococcus aureus.*

| **Group** | **Sample** | **Culture** | **PCR viral tests** | **Antibiotic regimen at Day 2** |
| --- | --- | --- | --- | --- |
| mPCR | sputum | negative | Herpex Simplex Virus on NPS | cotrimoxazole rovamycin cefepime |
| control | sputum | >10^7 *Pseudomonas aeruginosa* WT  >10^7 *Enterobacter cloacae* Amp-C HP |  | piperacillin-tazo |
| control | sputum | negative |  | piperacillin-tazo |
| mPCR | sputum | negative |  | piperacillin-tazo |
| mPCR | ETA | negative |  | meropenem |
| control | BAL | negative |  | meropenem |
| control | ETA | 10^5 *Corynebacterium striatum* | SARS-CoV-2 on NPS | piperacillin-tazo |
| control | ETA | >10^7 *Pseudomonas aeruginosa* WT |  | linezolid cefepime |
| mPCR | ETA | negative |  | rifampicin piperacillin-tazo |
| control | sputum | >10^7 *Pseudomonas aeruginosa* WT |  | amoxiclav |
| control | BAL | >10^6 *Candida albicans* |  | meropenem vancomycin |
| mPCR | BAL | negative |  | meropenem line |
| mPCR | BAL | negative |  | meropenem vancomycin |
| mPCR | BAL | negative |  | cefiderocol |
| mPCR | sputum | >10^7 *Klebsiella pneumoniae* amoxicillin-clavulanate resistant |  | cefazolin |
| mPCR | sputum | negative |  | piperacillin-tazo |
| control | BAL | negative |  | cefepime |
| control | BAL | negative | SARS-CoV-2 on BAL | daptomycin rifampicin cefepime |
| control | BAL | >10^6 *Pseudomonas aeruginosa* WT  >10^6 SAMS |  | ceftazidime |
| control | BAL | >10^6 *Pseudomonas aeruginosa* WT  >10^6 *Enterococcus faecium*  10^5 *Enterobacter cloacae* AmpC HP  >10^6 *Klebsiella pneumoniae* AmpC | Rhinovirus on NPS | cotrimoxazole piperacillin-tazo linezolid |
| control | BAL | >10^6 *Klebsiella pneumoniae* penem resistant |  | cotrimoxazole amoxiclav |
| control | BAL | negative | Rhinovirus on BAL | cefepime |
| mPCR | aspi trach | negative |  | ceftazidime avibactam aztreonam daptomycin |
| mPCR | sputum | negative |  | piperacillin-tazo |
| control | Not performed |  | Coronavirus on NPS | ceftriaxone |
| control | ETA | negative |  | metronidazole cefazolin meropenem rovamycin piperacillin-tazo |
| control | ETA | 10^6 MSSA  10^4 *Serratia marcescens* WT |  | none |
| mPCR | sputum | >10^7 MSSA |  | ceftazidime |
| mPCR | BAL | negative |  | imipenem |
| mPCR | miniBAL | >10^6 MSSA  >10^6 *Escherichia coli* WT |  | cloxacillin |
| control | Not performed |  |  | piperacillin-tazo |
| control | BAL | negative |  | piperacillin-tazo cipro |
| control | BAL | negative | Coronavirus and SARS-CoV-2 on BAL | meropenem |
| mPCR | sputum | 10^6 *Acinetobacter baumannii* complex WT  10^6 *Enterobacter cloacae* WT |  | none |
| control | BAL | negative | Coronavirus and SARS-CoV-2 on NPS | meropenem |
| control | Not performed |  | Influenza virus on NPS | piperacillin-tazo |
| mPCR | BAL | negative | Coronavirus and SARS-CoV-2 on NPS | cefiderocol |
| control | BAL | >10^6 *Burkholderia cenocepacia.* ceftazidime susceptible |  | meropenem |
| mPCR | BAL | negative |  | cefepime metronidazole linezolid |
| control | sputum | negative |  | meropenem |
| mPCR | sputum | negative |  | imipenem |
| mPCR | BAL | negative |  | cefepime |
| control | sputum | negative |  | piperacillin-tazo |
| control | miniBAL | negative |  | meropenem |
| control | BAL | 10^5 *Klebsiella pneumoniae* WT |  | cefepime linezolid |
| mPCR | BAL | negative |  | cefepime |
| mPCR | sputum | negative |  | piperacillin-tazo |
| control | Not performed |  | Influenza virus on NPS | piperacillin-tazo |
| control | ETA | negative | SARS-CoV-2 on NPS | cotrimoxazole piperacillin-tazo |

# **Table S5****. Escalations and De-escalations.**

NA: Not applicable

| **Group** | **First modification of the antibiotic regimen**  Antibiotic regimen before > after change | **Time between inclusion and first modification (days)** | **De-escalation or escalation** | **Antibiotic discontinuation** | **Spectrum downgrading** |
| --- | --- | --- | --- | --- | --- |
| mPCR | ceftazidime > stop | 7 | De-escalation | yes | no |
| mPCR | cotrimoxazole spiramycin cefepime > stop | 6 | De-escalation | yes | no |
| mPCR | amoxiclav > stop | 6 | De-escalation | yes | no |
| control | cefotaxime metronidazole > amoxiclav | 0 | De-escalation | yes | yes |
| mPCR | piperacillin-tazobactam > piperacillin | 2 | De-escalation | no | yes |
| control | piperacillin-tazobactam > stop | 4 | De-escalation | yes | no |
| mPCR | piperacillin-tazobactam > stop | 3 | De-escalation | yes | no |
| control | amoxiclav > stop | 4 | De-escalation | yes | no |
| control | amoxiclav > stop | 5 | De-escalation | yes | no |
| control | cefepime > stop | 6 | De-escalation | yes | no |
| mPCR | cefepime > ceftriaxone | 1 | De-escalation | no | yes |
| mPCR | meropenem > stop | 18 | De-escalation | yes | no |
| control | meropenem > cefotaxime | 5 | De-escalation | no | yes |
| mPCR | amoxiclav > stop | 6 | De-escalation | yes | no |
| control | piperacillin-tazobactam > stop | 3 | De-escalation | yes | no |
| control | linezolid cefepime > linezolid | 14 | De-escalation | yes | no |
| mPCR | piperacillin-tazobactam > cefotaxime | 0 | De-escalation | no | yes |
| control | cefotaxime spiramycin > cefotaxime | 0 | De-escalation | yes | no |
| control | piperacillin-tazobactam spiramycin > spiramycin | 2 | De-escalation | yes | no |
| control | amoxiclav > stop | 7 | De-escalation | yes | no |
| mPCR | piperacillin-tazobactam > ceftazidime | 2 | De-escalation | no | yes |
| mPCR | piperacillin-tazobactam > amoxiclav | 0 | De-escalation | no | yes |
| control | meropenem vancomycin > stop | 6 | De-escalation | yes | no |
| mPCR | meropenem linezolid > linezolid | 6 | De-escalation | yes | no |
| control | cefepime metronidazole > cefepime | 1 | De-escalation | yes | no |
| mPCR | meropenem vancomycin > meropenem | 3 | De-escalation | yes | no |
| mPCR | cefiderocol > stop | 10 | De-escalation | yes | no |
| mPCR | amoxiclav > stop | 6 | De-escalation | yes | no |
| mPCR | piperacillin-tazobactam > stop | 6 | De-escalation | yes | no |
| control | cefepime > amoxiclav | 4 | De-escalation | no | yes |
| control | daptomycin rifampicin cefepime > daptomycin rifampicin | 4 | De-escalation | yes | no |
| mPCR | cefotaxime > stop | 7 | De-escalation | yes | no |
| mPCR | cefepime > amoxiclav | 0 | De-escalation | no | yes |
| control | cotrimoxazole piperacillin-tazobactam linezolid > cotrimoxazole ceftazidime linezolid | 2 | De-escalation | no | yes |
| control | cefepime > amoxiclav | 5 | De-escalation | no | yes |
| control | cefepime > amoxiclav | 5 | De-escalation | no | yes |
| mPCR | imipenem > ceftriaxone | 0 | De-escalation | no | yes |
| mPCR | imipenem > stop | 16 | De-escalation | yes | no |
| control | cefepime > amox | 2 | De-escalation | no | yes |
| mPCR | piperacillin-tazobactam > stop | 7 | De-escalation | yes | no |
| control | ceftriaxone > stop | 6 | De-escalation | yes | no |
| control | piperacillin-tazobactam > stop | 1 | De-escalation | yes | no |
| mPCR | ceftazidime amika > ceftazidime | 2 | De-escalation | yes | no |
| control | cefotaxime > stop | 7 | De-escalation | yes | no |
| mPCR | méro > cefotaxime | 0 | De-escalation | no | yes |
| mPCR | imipenem linezolid > imipenem | 2 | De-escalation | yes | no |
| mPCR | ceftaroline clindamycin > cloxacillin | 1 | De-escalation | yes | no |
| control | piperacillin-tazobactam spiramycin > piperacillin-tazobactam | 0 | De-escalation | yes | no |
| mPCR | ciprofloxacin cefepime > cefotaxime | 0 | De-escalation | yes | yes |
| mPCR | cefepime > cefotaxime | 0 | De-escalation | yes | no |
| mPCR | cefotaxime spiramycin > stop | 2 | De-escalation | yes | no |
| control | piperacillin-tazobactam spiramycin > cefotaxime | 1 | De-escalation | yes | yes |
| control | meropenem > stop | 7 | De-escalation | yes | no |
| mPCR | imipenem doxycyclin colistin > stop | 2 | De-escalation | yes | no |
| control | meropenem > stop | 3 | De-escalation | yes | no |
| control | piperacillin-tazobactam > amox | 0 | De-escalation | no | yes |
| control | mero > cefotaxime | 1 | De-escalation | no | yes |
| mPCR | cefiderocol > stop | 21 | De-escalation | yes | no |
| control | meropenem amika vancomycin > meropenem | 0 | De-escalation | yes | no |
| control | amikacin vancomycin > stop | 0 | De-escalation | yes | no |
| mPCR | cefotaxime > stop | 6 | De-escalation | yes | no |
| control | cefepime > stop | 8 | De-escalation | yes | no |
| control | cefepime > cefotaxime | 2 | De-escalation | no | yes |
| mPCR | imipenem > cefotaxime | 4 | De-escalation | no | yes |
| control | piperacillin-tazobactam > cefotaxime | 1 | De-escalation | no | yes |
| mPCR | meropenem vancomycin > levofloxacin | 1 | De-escalation | yes | yes |
| mPCR | amoxiclav > stop | 4 | De-escalation | yes | no |
| mPCR | imipenem > stop | 0 | De-escalation | yes | no |
| control | piperacillin-tazobactam amika > stop | 1 | De-escalation | yes | no |
| mPCR | cefepime linezolid > cefepime | 1 | De-escalation | yes | no |
| control | piperacillin-tazobactam > stop | 6 | De-escalation | yes | no |
| mPCR | cefepime > stop | 7 | De-escalation | yes | no |
| mPCR | cefepime > cefotaxime | 0 | De-escalation | no | yes |
| mPCR | cefepime linezolid > cefotaxime | 0 | De-escalation | yes | yes |
| control | cefepime linezolid > stop | 7 | De-escalation | yes | no |
| control | cefepime > amoxiclav | 1 | De-escalation | no | yes |
| control | cefepime > amoxiclav | 1 | De-escalation | no | yes |
| mPCR | cefepime > amoxiclav | 3 | De-escalation | no | yes |
| mPCR | piperacillin-tazobactam > stop | 3 | De-escalation | yes | no |
| mPCR | piperacillin-tazobactam > stop | 2 | De-escalation | yes | no |
| control | meropenem > stop | 2 | De-escalation | yes | no |
| mPCR | meropenem > stop | 7 | De-escalation | yes | no |
| control | piperacillin-tazobactam spiramycin > piperacillin-tazobactam | 0 | De-escalation | yes | no |
| control | cotrimoxazole piperacillin-tazobactam > piperacillin-tazobactam | 6 | De-escalation | yes | no |
| control | meropenem > stop | 2 | De-escalation | yes | no |
| control | cefotaxime metronidazole > piperacillin-tazobactam | 0 | Escalation | NA | NA |
| control | amoxiclav > piperacillin-tazobactam cefazolin | 3 | Escalation | NA | NA |
| mPCR | cefazolin > ceftazidime | 3 | Escalation | NA | NA |
| mPCR | ceftriaxone > ceftazidime | 1 | Escalation | NA | NA |
| mPCR | ceftazidime-avibactam aztreonam > ceftazidime-avibactam aztreonam daptomycin | 1 | Escalation | NA | NA |
| control | metronidazole cefazolin meropenem > metronidazole cefazolin meropenem spiramycin piperacillin-tazobactam | 1 | Escalation | NA | NA |
| mPCR | piperacillin-tazobactam amika > meropenem amikacin | 0 | Escalation | NA | NA |
| control | cefotaxime > piperacillin-tazobactam ciprofloxacin | 2 | Escalation | NA | NA |
| mPCR | cefotaxime > piperacillin-tazobactam | 7 | Escalation | NA | NA |
| control | amoxiclav > meropenem amikacin | 2 | Escalation | NA | NA |
| control | piperacillin-tazobactam > meropenem | 1 | Escalation | NA | NA |
| control | amox > cefotaxime | 0 | Escalation | NA | NA |
| control | cefotaxime > meropenem | 1 | Escalation | NA | NA |

# **Figure S6. Antibiotics at inclusion (empirical) and at day 2.**

N=108 patients (missing data in 1 patient).

**
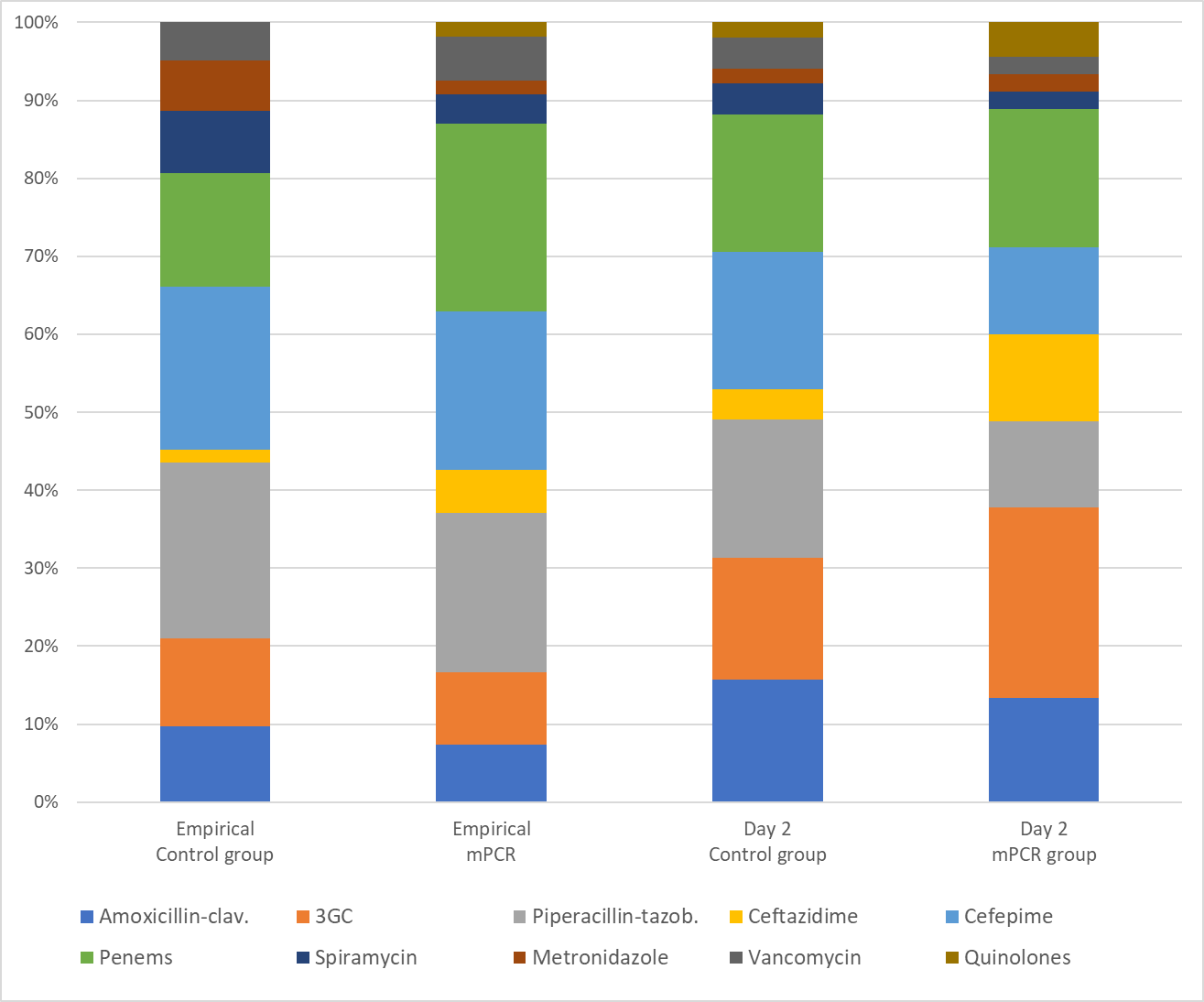
**

# **Table** **S7. Subgroup analysis - ICU**

Subgroup analysis of patients hospitalized in ICU at time of pneumonia onset (n=72). Data are n (%) or mean (standard error)

|  | mPCR group (N=34) | Control group (N=38) | Total (N=72) | p-value |
| --- | --- | --- | --- | --- |
| Vital status at day 30 or end of follow-up |  |  |  | 0.09^1^ |
| Alive | 34 (100%) | 35 (92%) | 69 (81%) |  |
| Dead | 0 (0%) | 3 (8%) | 3 (20%) |  |
| Adequate therapy on day of randomization | 20 (59%) | 9 (24%) | 29 (40%) | 0.002^1^ |
| De-escalation on day of randomization | 10 (29%) | 14 (37%) | 24 (33%) | 0.50^1^ |
| Escalation on day of randomization | 1 (3%) | 5 (13%) | 6 (8%) | 0.20^2^ |

^1^Chi-Square p-value ; ^2^Fisher exact test p-value

# **Table S8. Subgroup analysis – non-ICU wards**

Subgroup analysis of patients hospitalized in non-ICU wards at time of pneumonia onset (n=37). Data are n (%) or mean (standard error)

|  | mPCR group (N=21) | Control group (N=16) | Total (N=37) | p-value |
| --- | --- | --- | --- | --- |
| Transfer to the ICU | 3 (14%) | 0 | 0.13 | 0.62^1^ |
| Vital status at day 30 or end of follow-up |  |  |  | 0.72^2^ |
| Alive | 19 (90%) | 15 (94%) | 34 (89%) |  |
| Dead | 2 (10%) | 1 (6%) | 3 (11%) |  |
| Adequate therapy on day of randomization | 6 (29%) | 4 (25%) | 10 (27%) | 1^1^ |
| De-escalation on day of randomization | 4 (19%) | 1 (6%) | 5 (13%) | 0.36^1^ |
| Escalation on day of randomization | 0 | 2 (13%) | 2 (5%) | 0.18^1^ |

^1^Fisher exact test p-value; ^2^Chi-Square p-value

# **Table S9.** **Cost-consequence analysis.**

|  | mPCR group  n=55  (€) | Control group  n=54  (€) | Difference [95% Confidence Interval]  (€) |
| --- | --- | --- | --- |
| mPCR test | 133 | 0 | 133 |
| Laboratory time | 9 | 0 | 9 |
| Antimicrobials | 2,849 | 423 | 2,426 [100; 6,236] |
| Hospital stay | 20,644 | 22,379 | -1,735 [-7,054; 4,011] |
| Total cost | 23,635 | 22,802 | 833 [-5,227; 7,435] |

# **Figure S10. Tornado diagram**

One-way sensitivity analyses on the difference in total cost between the mPCR-guided strategy and the control group.


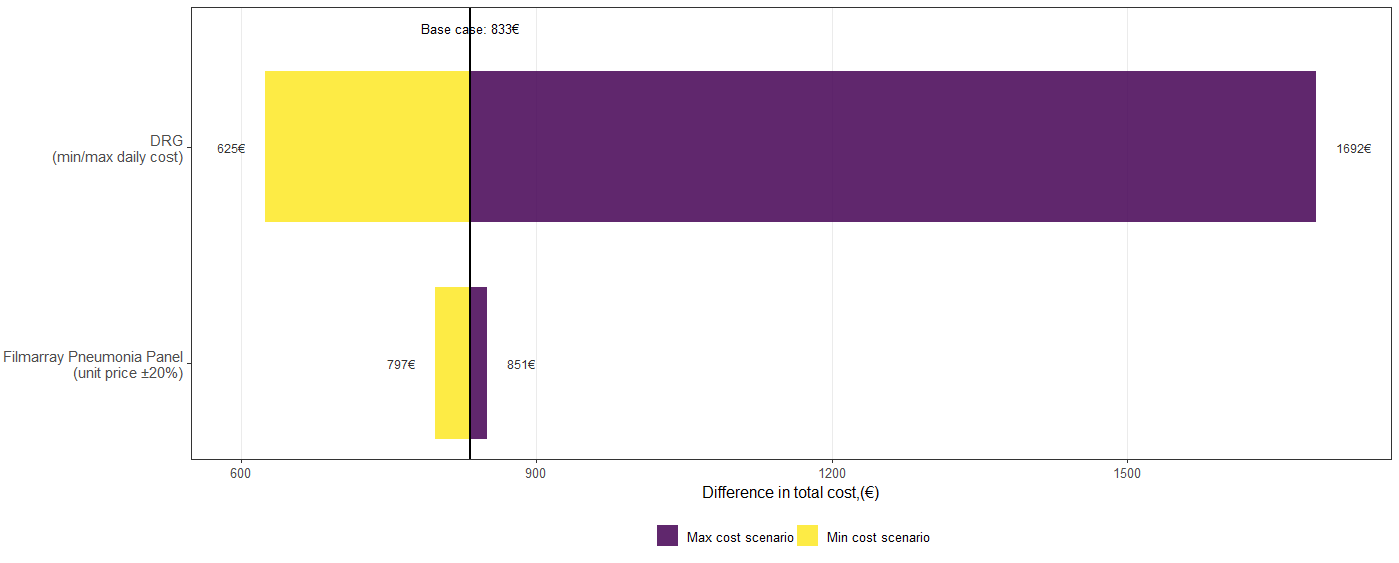

Supplement: dlag154_Supplementary_Data [file dlag154_supplementary_data.docx]
